# An Interesting Case of Sulphonylurea-Responsive Permanent Neonatal Diabetes Mellitus With Potassium Channel Mutation

**DOI:** 10.7759/cureus.108049

**Published:** 2026-04-30

**Authors:** Kristina O'Connell, Claudia Nadernejad, Caleb Bupp, Aditya Dewoolkar

**Affiliations:** 1 Chicago College of Osteopathic Medicine (CCOM), Midwestern University Chicago College of Osteopathic Medicine, Chicago, USA; 2 Neonatology, Helen de Vos Children's Hospital, Grand Rapids, USA; 3 Medical Genetics, Helen de Vos Children's Hospital, Grand Rapids, USA; 4 Endocrinology, Helen de Vos Children's Hospital, Grand Rapids, USA

**Keywords:** diabetes, hyperglycemia, hypothermia, neonatal care, whole genome sequencing

## Abstract

We present a case report of an infant diagnosed with a pathogenic de novo variant in *KCNJ11 (*Potassium Inwardly Rectifying Channel Subfamily J Member 11), associated with both transient and permanent neonatal diabetes. This patient is one of the earliest diagnosed and genetically confirmed patients with neonatal diabetes. Early detection is key, and this case report emphasizes the need for early consideration of rapid whole-genome sequencing as an option for diagnosis to significantly improve patient outcomes.

## Introduction

Neonatal diabetes is a rare but serious condition that affects numerous infants globally, with a reported incidence between 1:90000 and 1:160000 live births, and can result in significant complications if not promptly recognized and treated [1,2]. Permanent neonatal diabetes has been associated with activating variants in the genes encoding the two subunits of the ATP-sensitive potassium (KATP​) channel [3,4]. Among these, pathogenic de novo variants in the KCNJ11 gene (Potassium Inwardly Rectifying Channel Subfamily J Member 11) are a leading cause of both transient and permanent neonatal diabetes [3-5]. In addition, this variant is also linked with a spectrum of neurological features, including developmental delay and epilepsy, collectively termed DEND (Developmental delay, Epilepsy, and Neonatal Diabetes) syndrome [3,5].

The American Diabetes Association recommends genetic testing for all individuals diagnosed with diabetes before six months of age, as this allows for precise diagnosis and guides optimal management [6]. This recommendation aligns with the 2023 Laboratory Analysis Guidelines, emphasizing early molecular testing for monogenic diabetes to improve diagnostic accuracy [7]. Early detection, particularly through rapid whole genome sequencing (rWGS), is critical because it allows for timely identification of KCNJ11 variants and the potential to transition from insulin to oral sulfonylurea therapy [7,8]. A landmark international cohort study demonstrated that comprehensive genomic testing in neonatal diabetes significantly altered management in most cases, enabling precision treatment and improved outcomes [8].

Patients with KATP channel-related neonatal diabetes, including those with KCNJ11 variants, often achieve improved glycemic control with high-dose oral sulfonylureas, such as glyburide, compared to insulin therapy [9-12]. This treatment not only improves glycemic outcomes but may also improve neurological symptoms, especially when initiated early [10-12].

Glyburide exerts its effects by binding to the sulfonylurea receptor 1 (SUR1) subunit on the surface of pancreatic beta cells, forcing the KATP channel to close. This closure triggers a cascade of events that causes membrane depolarization and ultimately leads to insulin release into the bloodstream following a meal [13]. Initiating sulfonylurea therapy as early as possible yields the best glycemic and neurological results, though even delayed transition may still confer meaningful benefits [14,15]. Some experts have also discussed the merits and limitations of starting sulfonylureas empirically in suspected neonatal diabetes cases prior to confirmatory genetic results [12]. Thus, early genetic diagnosis, particularly using rapid whole-genome sequencing, plays a pivotal role in identifying actionable variants, initiating sulfonylurea therapy in a timely manner, and improving both glycemic and neurological outcomes [5,6,8-15].

## Case presentation

A male infant was delivered via induced vaginal delivery at 39 weeks' gestation due to a history of shoulder dystocia and clavicle fracture in a previously large-for-gestational-age infant. Maternal history was unremarkable, and prenatal labs were negative, including Group B Streptococcus, hepatitis B antigen, HIV, and rapid plasma reagin. Family history was significant for Congenital Hypothyroidism in the patient’s sister (due to a maternally inherited PAX8 gene variant).

In the delivery room, Apgar scores were 9 and 9 at 1 and 5 minutes, respectively, with no resuscitation interventions required. The infant received routine newborn care, including Hepatitis B vaccine, vitamin K injection, and erythromycin eye ointment. Hearing, critical congenital heart disease, and newborn screening were normal. Birth weight was 2.86 kg (11th percentile), length 49.5 cm (35th percentile), and head circumference 35 cm (61st percentile). Thyroid-stimulating hormone (TSH) and free thyroxine (FT4), checked on day one of life, were normal. Bilirubin was low risk, and blood glucose was elevated between 110 and 130 mg/dL at 24 hours of life. Pediatric endocrinology was contacted and recommended follow-up with the primary care physician, with instructions to be contacted if persistent hyperglycemia is seen at the clinic visit. The patient was discharged home in stable condition from the Mother Baby Unit.

On day of life 4, the patient presented to the primary care physician’s office for establishment of care. The infant was hyperglycemic (191 mg/dL) and hypothermic (34.1 °C), so was sent to the emergency department for further evaluation. In the ED, the patient was hypothermic (34.0 °C improving to 36.1 °C with warming) with a glucose of 178 mg/dL. A sepsis workup was initiated. Electrolytes were normal, ammonia level was 108 µmol/L, and repeat TSH returned within normal limits, consistent with the normal value obtained on day 1 of life. He was admitted to the NICU for further care. The differential diagnosis for hypothermia and hyperglycemia in this infant includes neonatal diabetes, sepsis, and inborn errors of metabolism.

Upon arrival to the NICU, the newborn had signs of dehydration and minimal subcutaneous fat on exam, although birth weight was inconsistent with a small-for-gestational age infant. The patient was alert, appropriately interactive, and appeared comfortable with no signs of distress.

The physical exam was normal: anterior fontanelle soft and flat, lungs clear, no murmur, abdomen soft, no hepatomegaly, and normal reflexes. Repeat labs in the NICU were reassuring except for continued hyperglycemia (127-229 mg/dL). The infant was breastfed 1.5-2 oz every 2 to 3 hours with normal urine and stool output.

Endocrinology was consulted, and beta-hydroxybutyrate, C-peptide, and ultrasound of the pancreas were ordered. Consultation with genetics was recommended. All results were unremarkable except for a low C-peptide level, which resulted after discharge. Neonatal diabetes was considered, and genetic testing for the various mechanisms was discussed. The patient was discharged home on day of life 6 with follow-up scheduled with endocrinology and instructions to monitor glucose levels at home. Outpatient genetic testing was planned, and insurance preauthorization was initiated.

Two days later, the patient required re-admission to the NICU for inpatient monitoring due to persistent hyperglycemia, with blood glucose levels exceeding 200 mg/dL at home. Because of the weekend, genetic testing had not yet been initiated. Glyburide was started as initial therapy at 0.05 mg/kg, and rapid whole genome sequencing was requested. This testing would allow fast results, interrogation for multiple potential genetic mechanisms, as well as clarification of the potential role of the familial PAX8 hypothyroidism. Preliminary rWGS returned prior to discharge, identifying the patient’s diagnosis and confirming correct treatment. The patient was discharged again on day of life 13 in stable condition. The Glyburide dosage at the time of discharge was 0.2 mg/kg at 0800, 0.3 mg/kg at 1600, and 0.2 mg/kg at 0000.

The infant is diagnosed with a pathogenic de novo variant in KCNJ11, associated with both transient and permanent neonatal diabetes. This diagnosis, particularly this patient’s gene variant, can be associated with developmental delay, seizures, permanent diabetes, and isolated permanent neonatal diabetes. No PAX8 variant was identified. This patient is currently responding to treatment with Glyburide and is meeting developmental milestones. This patient is one of the earliest diagnosed and genetically confirmed patients with neonatal diabetes. Early consideration of rWGS as an option for diagnosis could significantly improve outcomes. 

## Discussion

Neonatal diabetes mellitus (NDM) is a rare monogenic disorder most often presenting within the first six months of life, and its recognition has been transformed by advances in molecular genetics [3]. In contrast, infantile diabetes refers to diabetes diagnosed between 6 and 24 months of age and is more commonly autoimmune type 1 diabetes. Historically, infants with NDM were managed empirically with insulin therapy, as in autoimmune type 1 diabetes. However, it is now well-established that a large proportion of cases are caused by variants in the ATP-sensitive potassium (KATP​) channel genes, most commonly KCNJ11 and ABCC8 [4-7]. Genetic studies have greatly improved the understanding of the etiology of neonatal diabetes and led to more successful outcomes such as those presented in this case review. These genetic insights have not only clarified disease pathophysiology but have also revolutionized management by creating opportunities for targeted therapy. For this reason, the American Diabetes Association and multiple international groups now recommend that all infants diagnosed with diabetes before six months of age undergo genetic testing to confirm etiology and direct treatment decisions [6-8].

**Figure 1:**
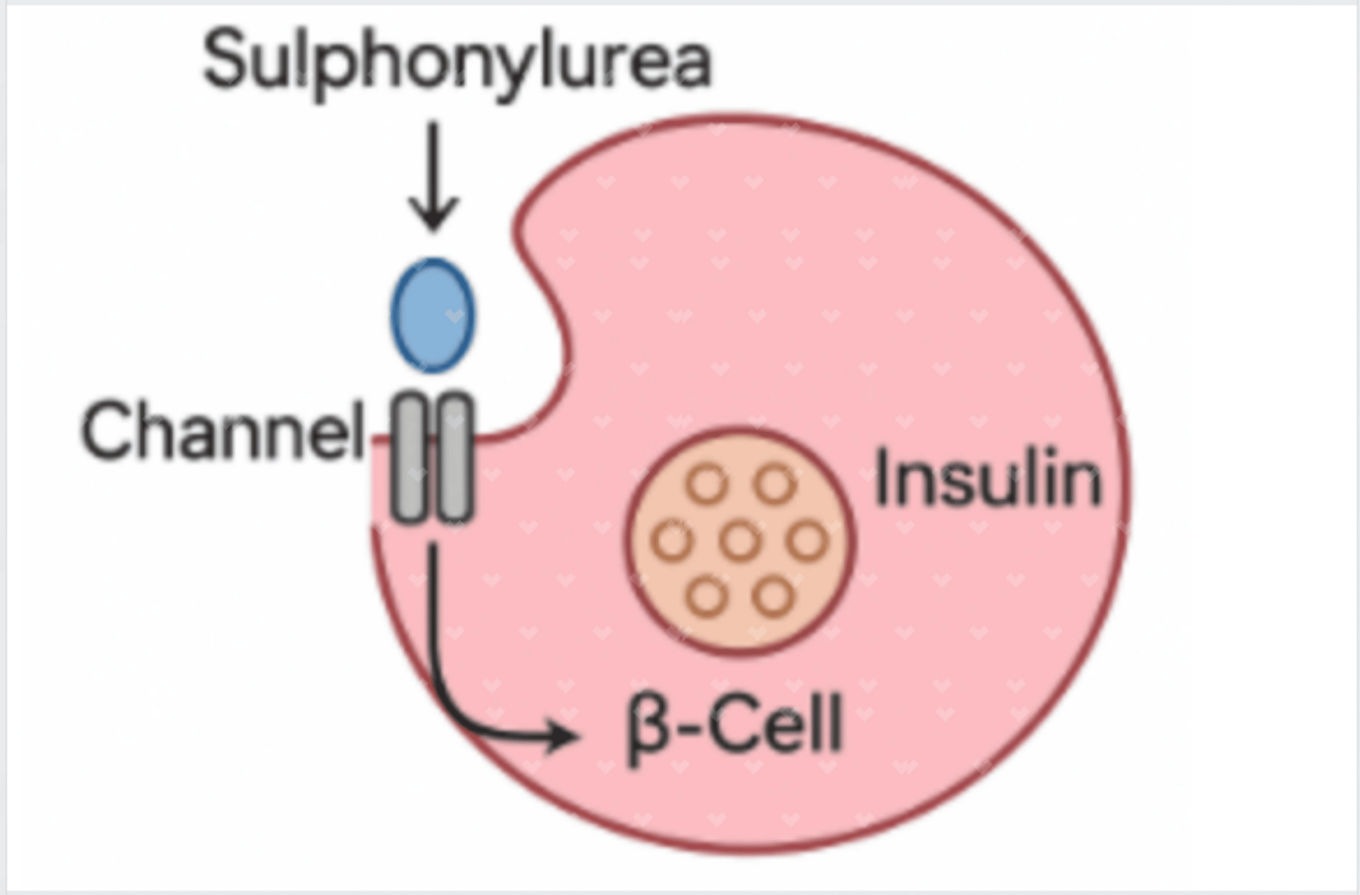
Mechanism of action of Glyburide

The KCNJ11 gene encodes the Kir6.2 pore-forming subunit of the KATP channel [4]. These variants lead to persistent opening of the KATP​ channel in pancreatic β-cells, preventing membrane depolarization and inhibiting insulin secretion [13]. Multiple reports of cases with diabetes due to KCNJ11 have been successfully treated with sulfonylureas such as glyburide, as presented in the case above [9-11]. These drugs act by maintaining closure of the KATP​ channels through direct binding to the subunit, allowing for optimal glucose control [13]. Sulfonylurea treatment has been shown to be more successful than insulin therapy alone; therefore, it is vital to test for genetic variants to determine the proper course of treatment [9,12,14,15]. Clinical studies have demonstrated that approximately 88% of patients with KCNJ11 variants can achieve improved glycemic control on sulfonylureas, with notable improvements in HbA1c and quality of life compared with insulin therapy [14-16]. Figure 1 shows the mechanism of action of Glyburide.

Studies demonstrate that activating variants in KCNJ11, encoding the Kir6.2 subunit of the KATP​ channel, account for a substantial proportion of both transient and permanent neonatal diabetes [4-6,11]. Due to the KATP​ channel’s distribution within the central nervous system, previous reports of KCNJ11 variants have been associated with developmental delay, epilepsy, and neonatal diabetes (DEND) [17]. Identifying DEND syndrome can be difficult because these neurological deficits tend to appear later in life [7,17]. Furthermore, the later treatment is begun, the less likely patients are to respond. Early initiation of sulfonylureas is associated with better neurodevelopmental outcomes, particularly in patients with DEND or intermediate DEND phenotypes [12,17]. Some variants (e.g., p.Val59Met, p.Ile296Leu) are more strongly associated with neurological manifestations, while others (e.g., p.Arg201His) may present with isolated diabetes, highlighting the importance of genotype-phenotype correlations in guiding care [4,5,8]. There is the option to empirically start sulfonylurea treatment prior to or in lieu of obtaining genetic testing, but the concern with this approach is that certain rare variants (e.g., p.Tyr330His) may confer sulfonylurea resistance, underscoring the importance of precise molecular diagnosis to guide therapy [18].

Although early precision treatment has not historically been common, ongoing research and broader access to genetic testing are making earlier diagnosis more achievable. Earlier identification allows for timely intervention, which may help prevent long-term diabetes-related complications. Moreover, establishing a specific genetic diagnosis enables individualized, targeted therapy and improves clinical outcomes, as demonstrated in this case. This patient represents one of the earliest genetically confirmed diagnoses of neonatal diabetes reported to date. His variant has been previously reported to be associated with DEND, but his treatment was initiated within two weeks of life, whereas many other cases targeted treatment is begun much later in life. At age three, he currently shows no signs of delay or seizures, and his diabetes is under appropriate control. Early detection is key, and this case report emphasizes the need for early consideration of rapid whole-genome sequencing to accelerate diagnosis and implement therapeutic changes, allowing for improved patient outcomes [8,9].

## Conclusions

Neonatal hyperglycemia is an abnormality that warrants further workup. Genetic causes, such as KCNJ11, should be considered in patients if they present with non-autoimmune and non-syndromic-related hyperglycemia. Consideration for rapid genetic testing is warranted. KCNJ11 variants are found in approximately 34% cases of those born with neonatal diabetes, and many of these individuals have successfully transitioned to sulfonylurea treatment from insulin therapy, resulting in optimal glucose control. Sulfonylurea treatment should be considered as a form of treatment in these patients.
